# Impact of Morphine Treatment on Infarct Size and Reperfusion Injury in Acute Reperfused ST-Elevation Myocardial Infarction

**DOI:** 10.3390/jcm9030735

**Published:** 2020-03-09

**Authors:** Ingo Eitel, Juan Wang, Thomas Stiermaier, Georg Fuernau, Hans-Josef Feistritzer, Alexander Joost, Alexander Jobs, Moritz Meusel, Christian Blodau, Steffen Desch, Suzanne de Waha-Thiele, Harald Langer, Holger Thiele

**Affiliations:** 1 University Heart Center Lübeck, Medical Clinic II (Cardiology/Angiology/Intensive Care Medicine), University Hospital Schleswig-Holstein, 23538 Lübeck, Germany; jwang1982@outlook.com (J.W.); thomas.stiermaier@uksh.de (T.S.); georg.fuernau@uksh.de (G.F.); alexander.joost@uksh.de (A.J.); moritz.meusel@uksh.de (M.M.); christian.blodau@uksh.de (C.B.); harald.langer@uksh.de (H.L.); 2 German Center for Cardiovascular Research (D.Z.H.K.), partner site Hamburg/Kiel/Lübeck, 23538 Lübeck, Germany; 3 The Second People’s Hospital of Yibin, 644000 Yibin, Sichuan, China; 4 Department of Internal Medicine/Cardiology, University of Leipzig–Heart Center, 04289 Leipzig, Germany; Hans-Josef.Feistritzer@medizin.uni-leipzig.de (H.-J.F.); Holger.Thiele@medizin.uni-leipzig.de (H.T.)

**Keywords:** morphine, infarct size, reperfusion, ST-elevation myocardial infarction, CMR

## Abstract

Current evidence regarding the effect of intravenous morphine administration on reperfusion injury and/or cardioprotection in patients with myocardial infarction is conflicting. The aim of this study was to evaluate the impact of morphine administration, on infarct size and reperfusion injury assessed by cardiac magnetic resonance imaging (CMR) in a large multicenter ST-elevation myocardial infarction (STEMI) population. In total, 734 STEMI patients reperfused by primary percutaneous coronary intervention <12 h after symptom onset underwent CMR imaging at eight centers for assessment of myocardial damage. Intravenous morphine administration was recorded in all patients. CMR was completed within one week after infarction using a standardized protocol. The clinical endpoint of the study was the occurrence of major adverse cardiac events (MACE) within 12 months after infarction. Intravenous morphine was administered in 61.8% (*n* = 454) of all patients. There were no differences in infarct size (17%LV, interquartile range [IQR] 8–25%LV versus 16%LV, IQR 8–26%LV, *p* = 0.67) and microvascular obstruction (*p* = 0.92) in patients with versus without morphine administration. In the subgroup of patients with early reperfusion within 120 min and reduced flow of the infarcted vessel (TIMI-flow ≤2 before PCI) morphine administration resulted in significantly smaller infarcts (12%LV, IQR 12–19 versus 19%LV, IQR 10–29, *p* = 0.035) and reduced microvascular obstruction (*p* = 0.003). Morphine administration had no effect on hard clinical endpoints (log-rank test *p* = 0.74) and was not an independent predictor of clinical outcome in Cox regression analysis. In our large multicenter CMR study, morphine administration did not have a negative effect on myocardial damage or clinical prognosis in acute reperfused STEMI. In patients, presenting early ( ≤120 min) morphine may have a cardioprotective effect as reflected by smaller infarcts; but this finding has to be assessed in further well-designed clinical studies

## 1. Introduction

Platelet inhibition by dual antiplatelet therapy with aspirin and a P2Y12 receptor inhibitor is the cornerstone of treatment for prevention of thrombotic events in patients with acute coronary syndrome (ACS) undergoing primary percutaneous coronary intervention (PPCI) [1,2]. The preferred P2Y12 inhibitors are prasugrel or ticagrelor due to the proven more rapid onset of action, greater potency, and superior clinical outcomes in comparison to clopidogrel [3,4,5].

In addition, co-administration of titrated intravenous opiates (e.g., morphine) is recommended by current guidelines (class IIa C recommendation) in patients with persisting severe chest pain to relieve angina, and to reduce associated sympathetic activation possibly causing vasoconstriction and increasing myocardial workload [1,2]. However, morphine inhibits gastric emptying, reduces intestinal motility, and induces nausea or vomiting [6]. These effects are associated with a slower uptake, delayed onset of action, and diminished effects of oral antiplatelet agents, which may lead to decreased peak plasma levels and subsequent early treatment failure [7]. In patients with ACS, morphine significantly decreased the plasma concentrations of ticagrelor and its active metabolite with subsequent impaired inhibition of platelets [7,8,9]. Moreover, the use of morphine was associated with higher mortality in patients with non-ST-elevation myocardial infarction (NSTEMI) [10], and with suboptimal reperfusion success after PPCI in patients with ST-elevation myocardial infarction (STEMI) [11].

In contrast, there is also evidence that opioid agonists may be involved in favorable cardioprotective effects on the myocardium, with beneficial effects on all major determinants of ischemia-reperfusion injury (infarction/apoptosis, arrhythmogenesis, contractile dysfunction, inflammation) [12,13,14]. However, previous studies reported inconsistent results regarding the effect of morphine on myocardial damage in patients with acute myocardial infarction [15,16,17,18] and had several limitations including single-center design, small sample size, and indirect infarct size assessment. Cardiac magnetic resonance (CMR) imaging is the reference standard technique for the assessment of infarct size and reperfusion injury [19].The aim of this study was to comprehensively evaluate the impact of morphine treatment on infarct size and reperfusion injury assessed by CMR in an adequately-sized multicenter study of patients with acute STEMI undergoing PPCI.

## 2. Materials and Methods

### 2.1. Study Design

This study was a predefined sub-study of the AIDA STEMI trial (*Abciximab Intracoronary versus Intravenously Drug Application in STEMI)* that compared intravenous versus intracoronary abciximab application in patients with STEMI and did not show any difference in infarct size, reperfusion injury, and clinical outcome between the treatment groups [20,21]. The detailed trial design, inclusion and exclusion criteria, as well as main results have been published previously [19,20,21,22]. Briefly, AIDA STEMI was a randomized, open-label, multicenter trial. Patients presenting with STEMI in the first 12 h after symptom onset were randomly assigned in a 1:1 ratio to intracoronary versus intravenous abciximab bolus administration (0.25 mg/kg bodyweight) during PPCI, with a subsequent 12-h intravenous infusion at 0.125 μg/kg per minute (maximum 10 μg/min). Patients were enrolled at 22 sites in Germany, with a final trial population of 2065 patients (intracoronary abciximab (*n* = 1032) and intravenous abciximab (*n* = 1033)). The study was approved by national regulatory authorities and ethics committees of participating centers. All patients provided written informed consent.

Consecutive patients enrolled in the AIDA STEMI trial at 8 sites were included in the CMR sub-study [21]. The sites were chosen based on proven expertise in performing CMR examinations in patients with acute myocardial infarction. By protocol, CMR was performed on days 1 to 10 after the index event for the assessment of myocardial salvage, infarct size, presence and extent of microvascular obstruction, left ventricular (LV) ejection fraction, and end-systolic and end-diastolic volumes. Intravenous morphine treatment before or during PPCI was prospectively recorded and patients were categorized as morphine treated or as patients without morphine treatment. The decision of whether patients should be treated with morphine or not was made by the emergency physician and/or the treating cardiologist.

### 2.2. Cardiac Magnetic Resonance Imaging

Patients underwent CMR imaging on a 1.5 or 3.0 T scanner (Siemens Magnetom Verio (3 T), Siemens, Germany; Siemens Avanto (1.5 T), Siemens; Siemens Symphonie (1.5 T), Siemens; Phillips Intera, CV (1.5 Tesla), Philips Medical Systems, The Netherlands; GE Signa Excite (1.5 T), General Electric, USA) between days 1 and 10 after infarction. The standardized imaging protocol and post-processing have been described elsewhere [21,23]. Cine sequences were used for the measurement of LV function and volumes, T2-weighted imaging for the assessment of the area at risk (AAR), and late enhancement imaging for the determination of infarct size (IS) and microvascular obstruction (MVO). Image analysis was performed by blinded personnel at the CMR core laboratory (University of Leipzig—Heart Center Leipzig, Leipzig, Germany).

Reproducibility and inter- as well as intra-observer variabilities of the CMR core laboratory were reported previously [22]. The measurements of AAR, IS, and MVO were expressed as the percentage of LV volume (% LV). Myocardial salvage index (MSI) was quantified from AAR and IS as described previously [24,25].

### 2.3. Clinical Outcome

The clinical endpoint of the morphine sub-study was the time to major adverse cardiac events (MACE), defined as the time from randomization to the occurrence of the composite of all-cause death, nonfatal re-infarction, and new congestive heart failure at 12 months after infarction. Detailed endpoint definitions are reported elsewhere [23]. Events were adjudicated by a clinical events committee blinded to the treatment assignment.

### 2.4. Statistical Analysis

Baseline patient characteristics, procedural details, and CMR findings are described according to the presence or absence of morphine administration. To adjust for potential confounders we performed additional analysis after matching for age ( +/− 3 years) and cardiovascular risk factors (hypertension, diabetes mellitus). All categorical variables were calculated as number and percentage of patients. Continuous variables were presented in medians and interquartile range (IQR). Differences between groups were analyzed with the Fisher exact test or the chi-square test for categorical variables and with the Student t test for continuous variables with normal distribution. In addition, the Wilcoxon rank-sum test was used for non-normally distributed continuous data.

The clinical endpoint was described by means of Kaplan–Meier curves and group differences were assessed by log rank test. Univariable Cox-regression analysis was performed for all variables of Table 1 to evaluate predictors of MACE within 12 months after randomization. All variables with a *p*-value <0.1 were considered as covariates in a stepwise multivariable Cox regression analysis. Within each iteration, the algorithm dropped the covariate with the highest *p*-value until all remaining covariates had a *p*-value < 0.05. Hazard ratios (HR) with 95% CI were calculated for binary outcomes. Statistical significance was considered as *p*-value < 0.05. We used SSPS version 22.0 for statistical analysis.

## 3. Results

**Figure 1:**
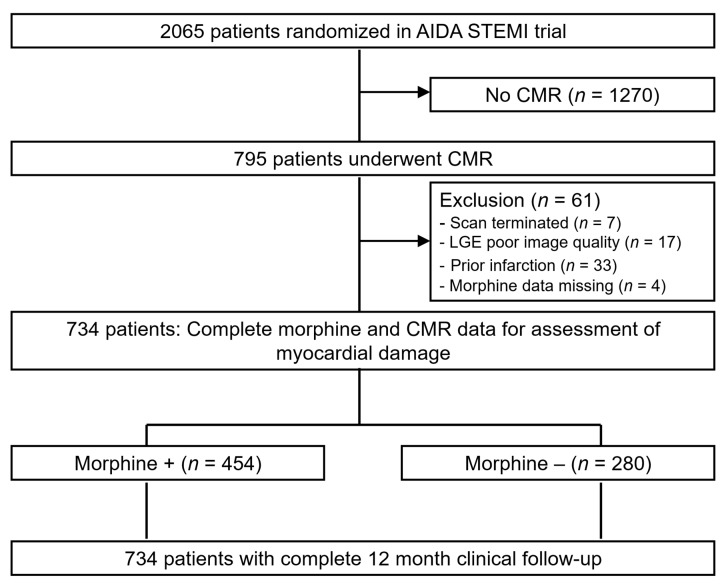
Study Flow. This study was a predefined sub-study of the AIDA STEMI trial (Abciximab Intracoronary Versus Intravenously Drug Application in STEMI). CMR = cardiac magnetic resonance, LGE = late gadolinium enhancement.

Morphine status was available in 791 patients (99.4%) (Figure 1). Patients with incomplete CMR scans (*n* = 7), poor image quality (*n* = 17), and prior infarction (*n* = 33) were excluded. Thus, the final study population consisted of 734 patients. Of these, 454 (61.5%) patients received intravenous morphine before or during PPCI versus 280 patients (38.5%) not receiving morphine.

### 3.1. Patient Characteristics

Demographic and clinical characteristics according to morphine treatment are shown in Table 1. The median age of the total enrolled study group was 62 years (IQR 51-71), and 555 patients (76%) were males. Patients receiving morphine were younger (*p* = 0.03), more often male (*p* = 0.02), and had a significantly lower incidence of hypertension (*p* = 0.02) and diabetes (*p* = 0.01) as compared to patients without morphine treatment.

The time from symptom onset to PCI hospital admission was also significantly shorter in the group of patients with morphine administration (*p* < 0.001). Compared with patients without morphine treatment, there was no significant difference in door-to-balloon time and other markers of angiographic (TIMI-flow pre-/post-PCI) or ECG (ST-segment resolution) reperfusion success.

### 3.2. CMR Parameters

The median time between the index event and CMR was three days (IQR 2–4) for both groups. The main findings from CMR analyses are displayed in Table 2. In all patients, the median infarct size was 17%LV (IQR 8 to 25) with no significant differences between the two groups (17% versus 16%, *p* = 0.67). Despite patients with morphine administration having larger LV end-diastolic volumes (145 mL (IQR 124–174) vs. 141 mL (IQR 112–166), *p* = 0.004), there was no difference in LV ejection fraction between groups. AAR, myocardial salvage, as well as microvascular obstruction were also similar between groups (all *p* > 0.05, Table 2).

To adjust for potential confounders, we performed matching for age +/– 3 years and were able to match 280 pairs of patients with versus without morphine administration (morphine group median age 66 years (interquartile range 51 to 73) versus no morphine group median age 66 years (interquartile range 52 to 72 years, *p* = 0.58)). However the results were not different as compared to the unmatched cohort for infarct size (morphine group: 16%LV (interquartile range 8 to 26%LV) versus no morphine group 17%LV (interquartile range 9 to 24%LV), *p* = 0.75) and other markers of myocardial damage (e.g., microvascular obstruction; *p* = 0.78). Moreover we performed matching for hypertension and diabetes status between groups (272 matched pairs). Again there was no difference in infarct size (morphine group: 17%LV (interquartile range 8 to 27%LV) versus no morphine group 17%LV (interquartile range 9 to 24%LV), *p*= 0.21) between patients treated with morphine versus without morphine treatment.

**Figure 2:**
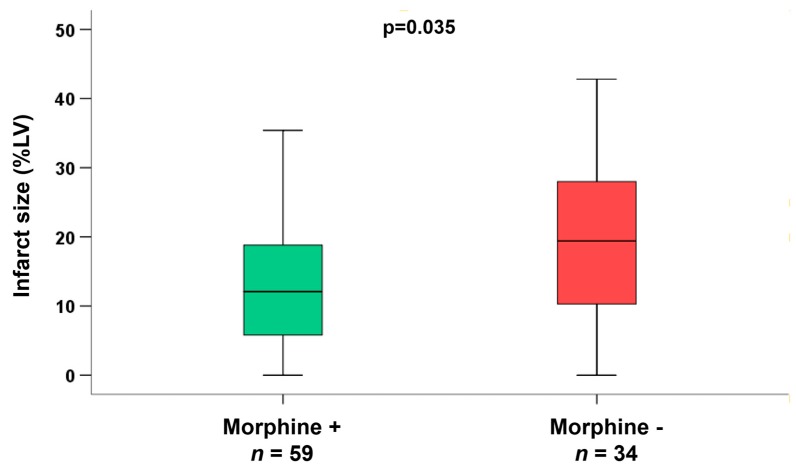
Infarct size in subgroup of patients with reperfusion within 120 min/2 h and TIMI-flow ≤2 before PCI. Observed a significantly reduced infarct size in the group of patients with morphine administration (*p* = 0.035).

**Figure 3:**
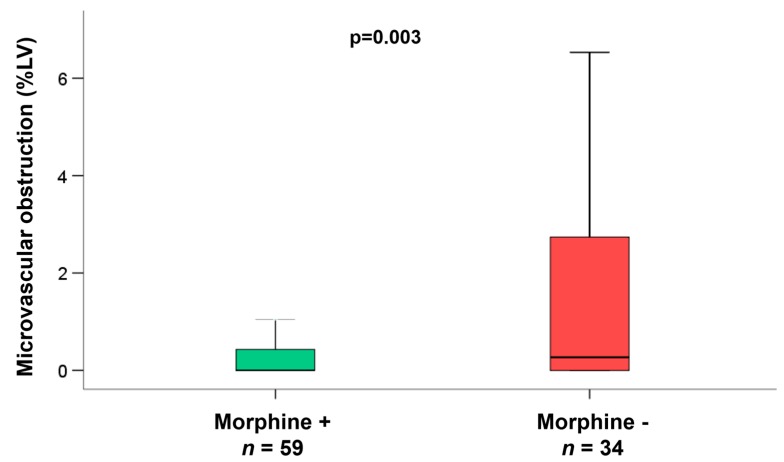
Microvascular obstruction in subgroup of patients with reperfusion within 120 min/2 h and TIMI-flow ≤2 before PCI. Observed a significantly smaller area of microvascular obstruction in the group of patients additionally treated with morphine (*p* = 0.003).

In the subgroup of patients (*n* = 93) with early reperfusion ( ≤ 120 min) and reduced flow of the infarcted vessel (TIMI-flow ≤2 pre-PCI), we observed a significantly reduced infarct size (*p* = 0.035, Figure 2), and a significantly smaller area of microvascular obstruction in the group of patients additionally treated with morphine (*p* = 0.003, Figure 3). Infarct size reduction was also evident in this subgroup when matching the patients for age ( +/− 3 years, morphine group 11%LV (interquartile range 5 to 18%LV) versus no morphine group 19%LV (interquartile range 10 to 29%LV), *p* = 0.027) and for cardiovascular risk factors (diabetes and hypertension; morphine group 12%LV (interquartile range 5 to 19%LV) versus no morphine group 19%LV (interquartile range 10 to 29%LV), *p* = 0.042). In patients with longer reperfusion times (reperfusion within 120 to 360 min *p* = 0.77 and reperfusion >360 min *p* = 0.40) there was no effect of morphine with respect to infarct size reduction.

### 3.3. Clinical Outcome

**Figure 4:**
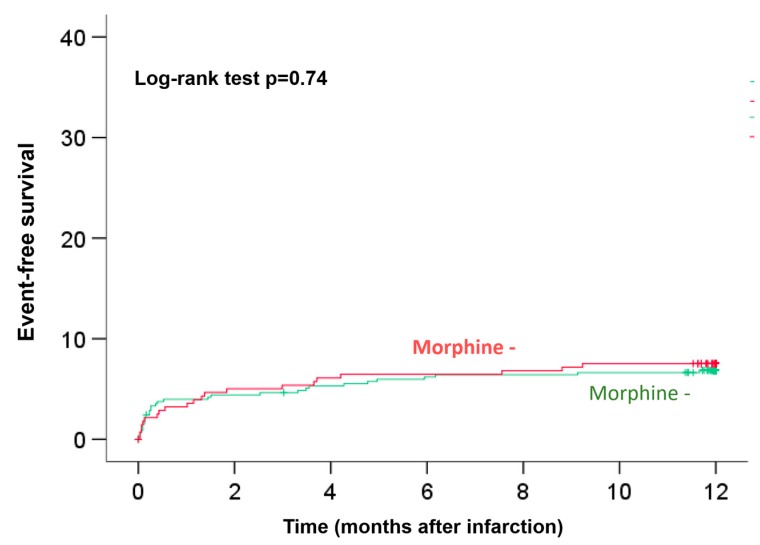
Twelve-month survival according to morphine use. Kaplan–Meier plots with log-rank testing for event-free survival at the 12-month follow-up. There were no significant differences between the two groups (Log-rank test *p* = 0.74).

Clinical 12-month follow-up was completed for all patients. Kaplan–Meier plots with log-rank testing demonstrated that there were no significant differences in the occurrence of event-free survival at the 12-month follow-up between the two groups (log-rank test p = 0.74, Figure 4). As most cardiovascular events occurred in the early phase after infarction, we performed a landmark analysis to get better insights regarding the effect of morphine and timing of hard clinical events. We performed 2 landmark analysis 1) after 30 days and 2) after two months (Figure S1A,B). Both analyses were consistent with the Kaplan–Meier curve after 12 months and did not identify a time period with differences between groups (30 days: HR 0.81 (95%CI 0.37–1.77), *p* = 0.60; one to 12 months: HR 1.52 (95%CI 0.68–3.41), *p* = 0.31 and two months: HR 1.14 (95%CI 0.57–2.27), *p* = 0.72; two to 12 months: 1.04 (95%CI 0.40–2.68); *p* = 0.94 (additional Kaplan–Meier Figures see Supplementary Materials).

Using stepwise multiple Cox regression, morphine administration was not a predictor of myocardial damage (infarct size (*p* = 0.35), microvascular obstruction, (*p* = 0.91)) and prognosis/clinical outcome (hazard ratio 1.10, 95% confidence interval 0.63 to 1.91, *p* = 0.74).

## 4. Discussion

To the best of our knowledge, the current study is the largest and the first multicenter investigation to comprehensively evaluate the impact of morphine administration on infarct size and reperfusion injury determined by CMR, the current reference standard for post infarction myocardial damage assessment. The main findings of our study can be summarized as follows: 1) Morphine administration is not associated with increased myocardial damage or impaired clinical outcome in STEMI patients undergoing primary PCI in association with Abciximab; 2) in the subgroup of patients with early reperfusion and reduced flow of the infarcted vessel morphine administration resulted in smaller infarcts and significantly less microvascular obstruction, indicating a potential cardioprotective effect of morphine administration in this subgroup of patients.

Morphine is a commonly-used drug in the acute phase of ACS to relieve pain, with the added potential benefit of attenuating acutely raised sympathetic tone. In current guidelines, morphine is recommended with decreasing strength of recommendation [1]. One reason is concern regarding a potentially significant interaction of morphine with antiplatelet agents. Previous studies have consistently shown that morphine use decreases the concentration and effects of P2Y12 inhibitors and is associated with reduced platelet inhibition [7,8,9]. However, the real clinical issue lies in whether the adverse effects of morphine on platelet inhibition are associated with an increased risk of stent thrombosis and worse clinical outcomes. In our large study there was no effect of morphine application on future cardiovascular events and myocardial damage. In contrast, other studies, including the large CRUSADE registry, demonstrated that intravenous morphine administration was associated with increased risk for clinical events in patients with NSTEMI, including higher in-hospital mortality, even after risk adjustment and propensity score matching. In STEMI patients the impact of morphine on reperfusion success and clinical outcome is still debated. The interaction between morphine and P2Y12 inhibitors has been proposed to explain, at least in part, the adverse outcomes, as demonstrated by impaired ST-segment resolution in patients with STEMI in the ATLANTIC trial [26]. On the other hand, in the FAST-MI 2010 cohort and in a sub-analysis of the CIRCUS trial, morphine was not associated with a higher risk of clinical events, including death and stent thrombosis [17,27]. The lack of randomized studies with clinical endpoints precludes drawing final conclusions regarding morphine use and clinically significant effects on patient outcomes. However, previous trials and our study may be limited by a selection bias of patients receiving morphine application because of more severe symptoms and presence of pulmonary edema. However, our study did not demonstrate different infarct characteristics regarding infarct severity, as demonstrated by no differences in Killip class at presentation and the amount of the myocardium at risk between groups.

The use of CMR allowed us to obtain further mechanistic insights into the impact of morphine on myocardial damage and reperfusion injury. Overall, there was no difference in infarct size and microvascular obstruction with respect to application or no application of morphine, thereby excluding a detrimental effect of morphine on reperfusion success in our study. Instead, in the subgroup of patients with early reperfusion and reduced flow of the infarcted vessel, CMR demonstrated smaller infarcts in morphine-treated patients. The effect of morphine in this subgroup may be explained by the enhanced effect of cardioprotection strategies when applied in the early phase of ischemia and most ischemic myocytes can be salvaged. Notably, in patients with prolonged ischemia we did not observe a morphine-induced infarct size reduction. Moreover, not only time to ischemia but also TIMI flow grade at admission in the infarct-related vessel was recognized as major determinant of myocardial injury. Patients with not completely occluded vessels (TIMI flow ≥I) may develop smaller infarct size, regardless of protection strategy and this may also influence the cardioprotective effect of morphine. These findings are in accordance with evidence that opioid agonists may be involved in favorable cardioprotective effects on the myocardium [13,14,15,16,17,18,28]. In animal models of myocardial reperfusion injury, morphine preconditioning improved cardiac function and reduced post-infarction remodeling [15,28,29,30]. It has been speculated that the cardioprotective effect of morphine as a pleiotropic drug is mediated via opioid receptors centrally [29] or via the activation of the PKCε-ERK1/2 pathway, thereby inhibiting mPTP opening [28]. Likewise, morphine may also activate P13K/Akt/eNOS signaling [31] and may involve, as a non-selective opioid agonist, not only μ-receptors [32] but also can combine and interact with κ- and δ-receptors [33,34]. Interestingly, κ-receptors played an advantageous role of ischemic preconditioning on infarct size [35,36,37] and arrhythmias [38,39] while δ-receptors were responsible for amelioration of infarct size only [37,39,40,41,42]. On the other hand, it has been suggested that morphine-induced attenuation of neutrophil and endothelial activation may be another way to induce cardioprotection in addition to significantly suppressed inflammatory responses, as assessed with IL-6, CD 11b, and CD 18 [12,43]. Such reduction of inflammatory response may; therefore, be also involved in cardioprotection and final infarct size reduction.

However, previous studies reported inconsistent results regarding the effect of morphine on myocardial damage in patients with acute myocardial infarction [15,16,17,18] and were mainly limited by preclinical studies, non-randomized observational study designs, as well as the assessment of indirect parameters of reperfusion success, like ST-segment resolution, TIMI flow grade after PPCI or infarct size measurement by biomarkers. Our results support a potential cardioprotective effect of morphine in patients with early reperfusion, but larger randomized studies are needed to finally elucidate the true clinical significance on hard clinical outcomes.

Our study has several limitations worth emphasizing. First, it was not a randomized trial and we cannot provide a power analysis for sample size calculation for our predefined sub-study. However, our study is by far the largest study assessing the effect of morphine on infarct size by use of CMR, the current reference standard for infarct size assessment. Second, the study was not powered to assess the effect of morphine on clinical endpoints and different patient characteristics of patients receiving morphine or not (e.g., patients receiving morphine had less comorbidity and had shorter time from symptom onset to PCI) might have influenced the results. Third, a main limitation of our study is the lack of data assessing interaction in terms of pharmacodynamics/kinetics of antiplatelet therapy, platelet function, and myocardial damage. Moreover, we cannot entirely rule out that the observed effects were, at least in part, mediated by metoclopramide, which is regularly co-administered with morphine. Finally, we could not analyze a dose-dependent effect of intravenous morphine on reperfusion success, as emergency physicians often did not document the exact doses applied. However, based on daily clinical practice, doses ranging from 5 to 10 mg can be assumed.

## 5. Conclusions

In our large, multicenter CMR study, morphine administration did not have an effect on myocardial damage or clinical prognosis in acute reperfused STEMI. In patients, presenting early (≤120 min) morphine may have a cardioprotective effect as reflected by smaller infarcts, but this finding has to be assessed in further well-designed clinical studies.

## Figures and Tables

**Table 1:** Patient Characteristics.

Variable		Total Study	Morphine +	Morphine −	*p*-Value
		*n* = 734	*n* = 454	*n* = 280	
Age (years)		62 (51–71)	61 (51–70)	66 (52–72)	0.03
Male sex: *n* (%)		555/734 (76%)	356/454 (78%)	199/280 (71%)	0.02
Cardiovascular risk factors: *n* (%)					
	Current smoking	316/670 (47%)	207/415 (50%)	109/255 (43%)	0.07
	Hypertension	488/731 (67%)	287/452 (64%)	201/279 (72%)	0.02
	Hypercholesterolemia	258/727 (36%)	156/450 (34%)	102/277 (37%)	0.55
	Diabetes mellitus	146/731 (20%)	76/453 (17%)	70/278 (25%)	0.01
BMI (kg/m^2^)		27.3 (24.8–30.1)	27.5 (25.0–30.2)	27.0 (24.6–30.1)	0.31
Anterior infarction: *n* (%)		343/702 (51%)	215/439 (49%)	128/263 (49%)	0.94
Heart-rate (min)		76 (67–87)	76 (66–86)	77 (70–88)	0.19
Systolic blood pressure (mmHg)		130 (117–147)	130 (116–145)	134 (119–150)	0.42
Diastolic blood pressure (mmHg)		80 (70–88)	80 (70–86)	80 (70–90)	0.82
Times (min)					
	Symptom onset to PCI hospital admission	180 (109–315)	165 (100–276)	200 (123–404)	<0.001
	Door-to-balloon-time	30 (22–42)	30 (21–40)	30 (23–45)	0.67
Killip-class on admission: *n* (%)					0.59
	1	650/734 (89%)	405/454 (89%)	245/280 (88%)	
	2	50/734 (7%)	28/454 (6%)	22/280 (8%)	
	3	17/734 (2%)	9/454 (2%)	8/280 (3%)	
	4	17/734 (2%)	12/454 (3%)	5/280 (2%)	
Number of diseased vessels: *n* (%)					0.76
	1	398/734 (54%)	251/454 (55%)	147/ 280 (53%)	
	2	206/734 (28%)	125/454 (28%)	81/280 (29%)	
	3	130/734 (18%)	78/454 (17%)	52/280 (19%)	
Infarct related artery: *n* (%)					0.69
	Left anterior descending	328/734 (45%)	199/454 (44%)	129/280 (46%)	
	Left circumflex	89/734 (12%)	56/454 (12%)	33/280 (12%)	
	Right coronary	314/734 (43%)	198/454 (44%)	116/280 (41%)	
	Left main	3/734 (0%)	1/454 (0%)	2/280 (1%)	
TIMI-flow before PCI: *n* (%)					0.47
	TIMI-flow 0	412/734 (56%)	246/454 (54%)	166/280 (59%)	
	TIMI-flow I	97/734 (13%)	66/454 (15%)	31/280 (11%)	
	TIMI-flow II	119/734 (16%)	75/454 (16%)	44/280 (16%)	
	TIMI-flow III	106/734 (14%)	67/454 (15%)	39/280 (14%)	
Thrombectomy: *n* (%)		111/454 (24%)	111/454 (24%)	68/280 (24%)	0.96
TIMI-flow post PCI: *n* (%)					0.17
	TIMI-flow 0	11/734 (1%)	7/454 (1%)	4/280 (1%)	
	TIMI-flow I	19/734 (3%)	13/ 454 (3%)	6/280 (2%)	
	TIMI-flow II	56/734 (8%)	27/454 (6%)	29/280 (10%)	
	TIMI-flow III	648/734 (88%)	407/454 (90%)	93/280 (86%)	
Peak CK (µmol/l*s)		26 (12–46)	27 (13–48)	26 (10–43)	0.28
ST-segment resolution (%)		55 (23–78)	58 (25–79)	51 (20–77)	0.12
Concomitant medications: *n* (%)					
	ß-blockers	703/732 (96%)	433/453 (96%)	270/279 (97%)	0.43
	ACE-inhibitors/AT-1-antagonist	698/732 (95%)	433/453 (96%)	265/279 (95%)	0.71
	Aspirin	734/734 (100%)	454 /454 (100%)	280 /280 (100%)	1
	Clopidogrel, prasugrel or both	734/734 (100%)	454 /454 (100%)	280 /280 (100%)	1
	Statins	699/732 (96%)	435/453 (96%)	264/279 (95%)	0.37
	Aldosterone antagonist	88/732 (12%)	51/453 (11%)	37/279 (14%)	0.42
	Completion of abciximab infusion	688/733 (94%)	429/453 (95%)	259/280 (93%)	0.94

Continuous data are presented as median and interquartile range. ACE = angiotensin-converting enzyme, AT-1 = angiotensin1, BMI = body mass index, CMR = cardiac magnetic resonance, CK = creatine kinase, PCI = primary percutaneous coronary intervention, TIMI = thrombolysis in myocardial infarction.

**Table 2:** Cardiovascular magnetic resonance results.

Characteristic	Total Study *n* = 734	Morphine + *n* = 454	Morphine − *n* = 280	*p*
Area at risk (edema) (%LV)	35 (25–48)	36 (25–48)	35 (27–48)	0.72
Infarct size (%LV)	17 (8–25)	16 (8–26)	17 (9–24)	0.67
Myocardial salvage (%LV)	17 (9–27)	17 (9–26)	17 (8–27)	0.45
Myocardial salvage index	51 (33–69)	51 (32–69)	52 (35–69)	0.65
Late MO (%LV)	0.0 (0.0–1.8)	0.0 (0.0–1.8)	0.0 (0.0 – 1.9)	0.92
LV ejection fraction (%)	51 (44–58)	51 (44–58)	50 (43–58)	0.71
LV end-diastolic volume (mL)	146 (121–171)	145 (124–174)	141 (112–166)	0.004
LV end-systolic volume (mL)	72 (54–91)	72 (55–93)	71 (52–88)	0.18

Continuous data are presented as median and interquartile range. CMR = cardiac magnetic resonance, LV = left ventricular, MO = microvascular obstruction.

## Supplementary Materials

The following are available online at https://www.mdpi.com/2077-0383/9/3/735/s1, Figure S1A and Figure S1B: Kaplan–Meier event curves with landmark analysis from 30 days (A) and two months (B) follow-up.
