# Supplementary material for: Impact of Morphine Treatment on Infarct Size and Reperfusion Injury in Acute Reperfused ST-Elevation Myocardial Infarction
Source: J Clin Med. 2020 Mar 9;9(3):735. doi: 10.3390/jcm9030735 (PMC7141264; doi:10.3390/jcm9030735)

Supplemental Figure 1A and 1B: Kaplan Meier Event Curves With Landmark Analysis from 30 days (A) and 2 months (B) follow-Up.

Figure 1A

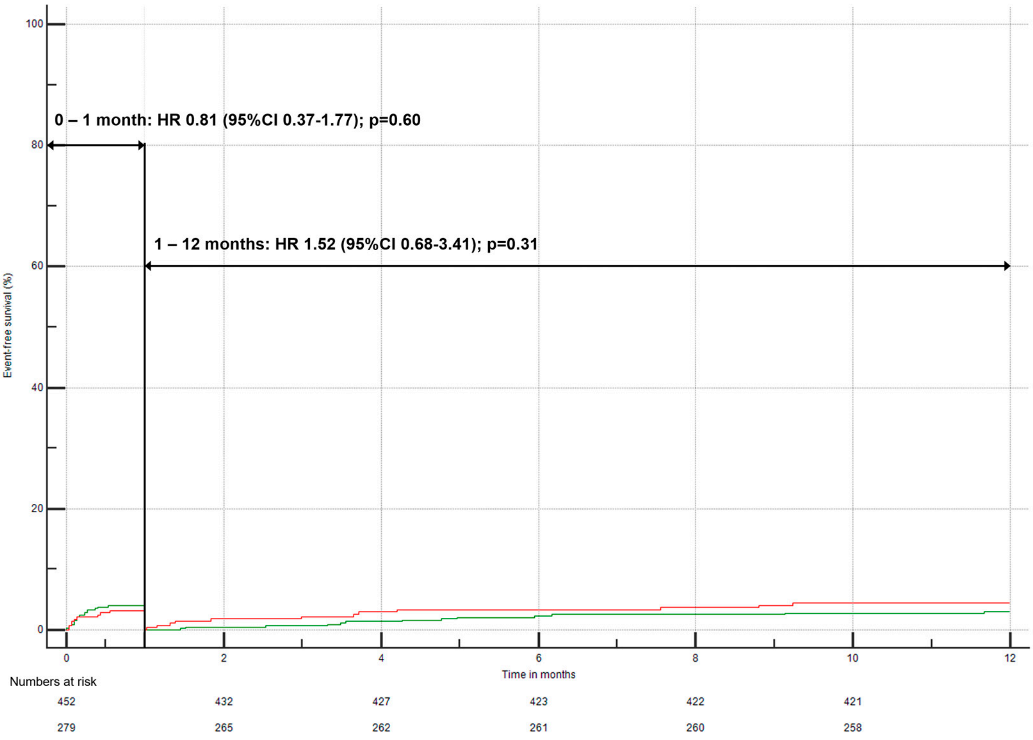

Figure 1B

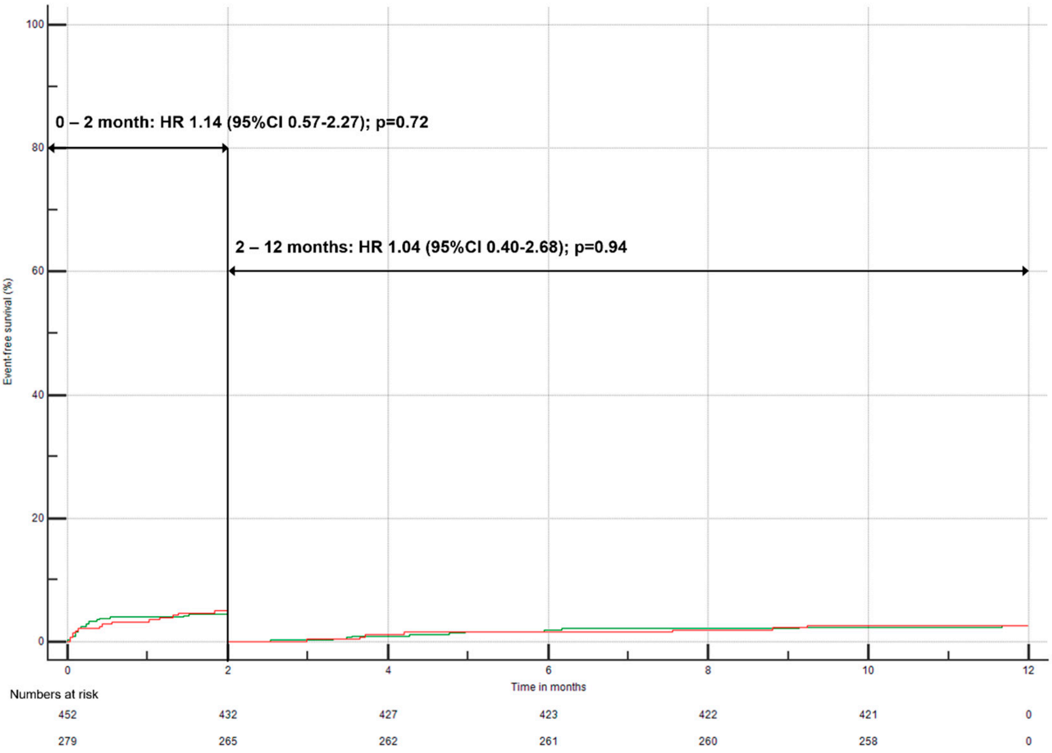

Supplement: Supplementary file 1 [file jcm-09-00735-s001.pdf]
